# Managerial competencies of hospital managers in South Africa: a survey of managers in the public and private sectors

**DOI:** 10.1186/1478-4491-6-4

**Published:** 2008-02-08

**Authors:** Rubin Pillay

**Affiliations:** 1 School of Business and Finance, University of the Western Cape, Private Bag X17, Bellville, 7535, South Africa

## Abstract

**Background:**

South Africa has large public and private sectors and there is a common perception that public sector hospitals are inefficient and ineffective while the privately owned and managed hospitals provide superior care and are more sustainable. The underlying assumption is that there is a potential gap in management capacity between the two sectors. This study aims to ascertain the skills and competency levels of hospital managers in South Africa and to determine whether there are any significant differences in competency levels between managers in the different sectors.

**Methods:**

A survey using a self administered questionnaire was conducted among hospital managers in South Africa. Respondents were asked to rate their proficiency with seven key functions that they perform. These included delivery of health care, planning, organizing, leading, controlling, legal and ethical, and self-management. Ratings were based on a five point Likert scale ranging from very low skill level to very high skill level.

**Results:**

The results show that managers in the private sector perceived themselves to be significantly more competent than their public sector colleagues in most of the management facets. Public sector managers were also more likely than their private sector colleagues to report that they required further development and training.

**Conclusion:**

The findings confirm our supposition that there is a lack of management capacity within the public sector in South Africa and that there is a significant gap between private and public sectors. It provides evidence that there is a great need for further development of managers, especially those in the public sector. The onus is therefore on administrators and those responsible for management education and training to identify managers in need of development and to make available training that is contextually relevant in terms of design and delivery.

## Background

South Africa has a dual health system characterized by large public and private sectors. The public sector serves the indigent population constituting more than 80% of the total and is funded predominantly by the government from general tax revenue, comprising 40% of total health spend. The private sector, which is highly developed, serves less than 20% of the population, comprising those who are insured or are high income earners, and is responsible for 60% of total health expenditure [1].

Hospitals in South Africa reflect the stark contrast between health service provision in the public and the private sectors. The government owned and managed public sector hospitals are often characterized as being inefficient and ineffective as evidenced by anecdotes of patient dissatisfaction and disaffections, and personal observation [2-4]. In contrast, the privately owned and managed hospitals are amongst the more profitable of enterprises and compare favourably with the best in the world, as evidenced by constantly rising share prices and the growth in medical tourism. In an attempt to improve public sentiment about the public sector, and in their quest to enhance efficiency and effectiveness within the sector, public sector agencies are aspiring to emulate the private sector philosophy and management approach. The underlying assumption is that there is a potential gap in management capacity between the two sectors. This study aims to ascertain the skills and competency levels of hospital managers in South Africa as determined by self assessment, and to find out whether there are any significant differences in proficiency levels between managers in the different sectors. The findings will therefore be useful in all countries where national health systems are making the transition to a more businesslike approach to service delivery.

All managers, irrespective of where or what they manage, perform four generic tasks. These include planning, organizing, leading and controlling. Planning involves defining goals and mapping out ways to reach them; organizing entails arranging and coordinating human, material and information resources aimed at achieving desired goals; leading involves motivating others to achieve organizational goals and controlling involves measuring performance and monitoring progress relative to objectives [5]. Managers need to possess several competencies that will enable them to perform these functions effectively and efficiently. Managerial competencies are 'sets of knowledge, skills, behaviours, and attitudes that a person needs to be effective in a wide range of managerial jobs and various types of organizations' [6]. Of particular importance are strategic skills which relate to setting of key objectives based on an understanding of what is happening inside and outside the organization; task related skills which encompass functional and operational competencies which enable one to define the best approach to achieving objectives, given the resources available; people related skills which enable one to achieve objectives through and with others; and self-management skills which enable one to take responsibility for one's life at work and beyond. The field of health care management, however, poses unique challenges as managers are expected to integrate modern business management practices with clinical and healthcare knowledge.

The American College of Preventive Medicine, after canvassing a broad audience of clinicians, educationists and health management experts, defined a list of health management competencies and performance indicators to assist in the development of training programs in medical management. These related to the delivery of health care, financial management, organizational management, and legal and ethical considerations [7]. In a survey of American medical directors, competencies related specifically to health care, including clinical preventive skills, were rated highly relative to generic management competencies, suggesting that a health background or specialized training in health care management may be essential for the effective management of health service organizations [8]. Similar studies in the UK, also identified financial, medical and people related skills as the most important for inclusion in management development programs for hospital managers [9,10].

Although health managers are key to overcoming the challenges facing health delivery in South Africa, there has been very little formal evaluation of the capacity of hospital managers, as well as their needs for future training, in South Africa. Schaay [11] emphasised the importance of determining the level of current management capacity and training required as part of an overall management development process in our quest to improve policy implementation and health systems functioning. This paper aims to evaluate hospital managers' perceptions about their developed abilities for their current role as well as their needs for further training in health care management. In addition it compares and contrasts the perceptions of hospital managers in the public sector with those in the private sector regarding these competencies and needs. It is hoped that the information gleaned will help in conceptualization, design and delivery of appropriate and relevant programs aimed at enhancing management and leadership capacity in the health sector in South Africa.

## Methods

Data for this study come from a survey of hospital managers in the public and private sectors in South Africa, using a self administered questionnaire. The survey was conducted among all managers of public hospitals in six of the nine provinces in South Africa (3 provinces did not respond to the call to participate) and all managers of private hospitals registered with the Hospital Association of South Africa, who represent 94% of private hospitals in the country. The final sample comprised 215 public sector managers and 189 private sector managers.

The survey instrument comprised a specially developed and pre-tested questionnaire that included biographic and institutional characteristics, professional background and exposure to health management training. Likert-type scales ranging from 1 (very poor) to 5 (excellent) and comprising multiple items, were used to rate their perceptions of their competency levels with seven key functions that they perform, as well as their needs for further training and development. These included specific health care competencies, planning, organizing, leading, controlling, legal and ethical, and self-management. These competencies were derived from the literature [[5-9], and [10]] thus ensuring content validity. They were found to exhibit high internal consistencies in their respective studies, thus ensuring the reliability of the instrument. Several other stakeholders with an interest in hospital management were asked to evaluate the relevance of the questionnaire to the proposed study objectives, thus establishing face validity.

Questionnaires were mailed in September 2006 and non-responders were sent questionnaires four weeks later. Data collection was terminated six weeks after the final mailing. The results of a sample of primary non-responders were compared to that of the primary responders to assess non-response bias. Data for individual variables were summarized using frequency distributions and focused on the central tendency (mean) and the dispersion (standard deviation). The ultimate score that each manager received for each of the seven factors was calculated from the mean of the summed items for that variable. This allows one to treat the data as interval data measuring a latent variable, to which parametric statistical tests can be applied. Reliability of scales was estimated by assessing the internal consistency of the scales using Cronbach's Alpha. Relationships between variables were analyzed using chi-squared tests for categorical variables. For quantifiable variables, the non-parametric Mann-Whitney was used for test for single item scales, and parametric analysis of variance (ANOVA) for responses that were summed to create a factor. All analyses were conducted at a 95% level of certainty and allowing for a margin of error of 5%.

## Results

Questionnaires were returned from 116 of 210 valid addresses of public sector managers and 87 of 179 valid addresses of private sector managers. This represents a total response rate of 51.91% and a response rate of 55.23% and 48.60% from the public and private sectors, respectively. There were no significant differences between primary respondents and the sample of primary non-respondents in terms of demographics, institutional characteristics and the self-evaluation of competency levels

As shown in Table 1, most public sector respondents were male (53.9%), between the ages of 35 to 50 (52.6%) and have been in their current positions for less than 5 years (50.4%). Private sector respondents were also predominantly male (62.1%) and between the ages of 35 and 50 (68.6%), but the majority (67.4%) have been in their positions for more than 5 years.

**Table 1:** Respondent Characteristic (Count and valid percentages)

	PUBLIC	PRIVATE
RESPONSE RATE	116(55.23)	87(48.60%)
Gender (N = 202)		
Male	62 (53.9%)	54 (62.1%)
Female	53 (46.1%)	33(37.9%)
Total	115	87
Age (N = 200)		
< 35	3 (2.7%)	6 (7.0%)
35–50	60 (52.6%)	59 (68.6%)
>50	51 (44.7%)	21 (24.4%)
Total	114	86
Years In Current Position (N = 200)		
<5	57 (50.5%)	28 (32.2%)
5–10	31 (27.4%)	39 (44.8%)
>10	25 (22.1%)	20 (23%)
Total	113	87
Primary Formal Qualification (N = 195)		
Medical/Health related	63 (55.3%)	28 (34.6%)
Commerce/General Management	14 (12.3%)	40 (49.4%)
Health Care Management	16 (14%)	1 (1.2%)
More than 1 of above 3	20 (17.5%)	6 (7.4%)
Other	1 (0.9%)	6 (7.4%)
Total	114	81
Formal Training In Health Management(N = 201)		
Yes	86 (74.8%)	36 (41.9%)
No	29 (25.2%)	50 (58.1%)
Total	115	86
Informal Training In Health Management (N = 202)		
Yes	108 (93.1)	77 (89.7%)
No	8 (6.9%)	9 (10.3%)
Total	116	86

Most public sector managers had a medical/health related background (55.3%) while the majority of managers in the private sector had a commerce/management or some other background (67.2%). Formal training in health management, in the form of a certificate, diploma or degree was higher among public sector managers (74.8%), relative to their private sector colleagues (41.9%). Both groups reported equally high levels of informal training in health management (around 90%). These included mentoring, in-service training and non-certified programs.

Bivariate analyses between the sectors and other categorical variables show significant associations between the sectors and age, experience, professional background, formal training in health management and the intention of respondents to attend future training programs in health care management (Table 2).

**Table 2:** Bivariate Relationship between Sectors and Respondent Biographic and Training Characteristics (Chi-square)

Other Biographics	Pearson Chi-squared	df	Significance
Gender	1.348	1	0.246
Age	9.780	2	0.008
Number of years in currency position	8.121	2	0.017
Primary formal qualification	41.916	4	0.000
Formal training	22.355	1	0.000
Informal training	1.492	2	0.474
Intention for further training	10.930	1	0.001

Public sector managers were more likely to be older than 50 years than their colleagues in the private sector

(p 0.008). Managers in the public sector were also significantly more likely to have less than five years experience while their private sector colleagues were more likely to have over five years experience

(p 0.017). Private sector managers were also more likely to have a commerce or management background while those in the public sector were more likely to have a health or medical background (p 0.000). Private sector managers were also significantly less likely to have had any formal training in health care management than their public sector colleagues (p 0.000) and were also less likely to be seeking any training in health management within the next five years (p 0.001).

The Cronbach's alpha and the mean total scores for the management competency subscales are presented in Table 3 below. The Cronbach's alphas for all the scales are at an acceptable level of reliability, averaging 0.845.

**Table 3:** Reliability of Management Competency and Need for Further Training Sub-Scales

	N of Items	Cronbach's Alpha	Mean total score
Competency Variables			
Specific health care skills	9	0.854	3.44
Planning	5	0.906	4.14
Organizing	7	0.865	3.90
Leading	6	0.888	4.02
Control	6	0.829	3.90
Legal and ethical issues	4	0.772	3.67
Self Management	6	0.801	4.04

As a group, hospital managers in South Africa perceive themselves as at least reasonably competent in all facets of their management functions (means > 3). They did however feel relatively more competent in their ability to plan (4.14), manage themselves (4.04) and lead (4.02) whilst feeling least competent in the specific health care skills (3.44).

Public sector managers reported that they felt most competent in planning (3.96), self management (3.95) and leading(3.91) and least competent in terms of legal/ethical competencies (3.59) and their specific health care skills (3.59). Private sector managers rated themselves highest in planning(4.30), followed by organizing(4.18), leading (4.16), self management (4.16) and controlling (4.14). They rated themselves as being least competent in legal/ethical skills (3.77) and their specific health care skills (3.21).

In total, 94.9% of public sector managers and 80.5% of private sector managers agreed with the statement that they "need further management development", suggesting that, on average, hospital managers felt that they could benefit from further training. The Mann-Whitney test revealed that public sector managers were also significantly more likely to report that they required further development and training than their private sector colleagues (Z = -6.441; p 0.00)

Parametric ANOVA revealed significant differences between the sectors and all of the management competency variables as well as the 'need for management development' (See Table 4).

**Table 4:** Bivariate Relationship between Sectors and Management Competency and Need for Further Training Variables (ANOVA)

		**N**	**Mean**	**F**	**Sig.**
**Competency Variables**					
Specific Health Care skills	Public Sector	111	3.59	19.859	0.000
	Private Sector	73	3.21		
	Total	184	3.44		
Planning	Public Sector	115	3.96	18.306	0.000
	Private Sector	85	4.30		
	Total	200	4.11		
Organizing	Public Sector	111	3.69	42.294	0.000
	Private Sector	84	4.18		
	Total	195	3.90		
Leading	Public Sector	114	3.91	10.910	0.001
	Private Sector	86	4.16		
	Total	200	4.02		
Control	Public Sector	116	3.72	34.621	0.000
	Private Sector	81	4.14		
	Total	197	3.89		
Legal and Ethical Issues	Public Sector	113	3.59	5.191	0.024
	Private Sector	87	3.77		
	Total	200	3.67		
Self Management	Public Sector	116	3.95	8.400	0.004
	Private Sector	87	4.16		
	Total	203	4.04		

Managers in the private sector perceived themselves to be significantly more competent than their public sector colleagues in all of the factors except for the "delivery of health care" where public sector managers felt more competent (p 0.009).

## Discussion

Our response rate, though modest, is good given that response rates in studies of professionals using self-administered questionnaires are generally poor [12,13]. This may indicate that managers attach a high degree of importance to medical management and the need for management development. The similarity in responses between respondents and a sample of primary non-responders suggests that non response bias was minimal and the sample was therefore representative of all hospital managers in South Africa.

The professional background of the respondents suggests that there is a perceptible shift in the leadership of hospitals in South Africa towards general managers with a background in commerce or management. Historically, all hospitals, especially in the public sector, were managed by medical superintendents and this shift is in keeping with a strategy aimed at improvement of sustainable and effective delivery of health services by strengthening management capacity [14]. However, although it is understandable in terms of the government's transformation policy, it is of concern that the majority of public sector managers are vastly inexperienced, having been in management positions for less than five years. In addition, the fact that public sector managers were significantly more likely to be over 50 years of age has implications in terms of the natural attrition and the replacement of these managers and on the return on investment from the development of these managers. The future sustainability and stability of public sector institutions will therefore depend not only on enhancing current management capacity, but also on the development of individuals with management potential as part of a broader career management and succession planning initiative.

The fact that public sector managers have rated themselves as at least "reasonably competent but not good" in all of the competencies suggests that they lack confidence in their ability either because they don't possess the requisite management skills or because they more generally lack self-belief. Either way, this is a potential hindrance to the reconceptualisation of the public sector into a more customer-oriented service and suggests that public sector managers need more training in management skills. In contrast their private sector colleagues have rated themselves as being "good to very good" in most of the competencies – especially those that relate to the core management functions of planning, organizing, leading, controlling and self-management. This suggests greater self confidence and perceived ability, which augurs well for the effective and efficient management of institutions in this sector.

The fact that there is a significant gap in competency levels of managers between the different sectors, with private sector managers rating themselves significantly higher on all of the competencies except for health services delivery, may partly explain the differences in performance between the sectors. This may be partly attributed to the predominance of managers with a management background in the private sector. Hospital managers are of the opinion that a management background is more appropriate for hospital management and those with a background in management tended to rate themselves as being more competent than their colleagues who had a clinical background [15]. The fact that private sector managers tended to be more experienced may also contribute to their perceived greater competency.

It is however of concern that despite public sector managers reporting that they were significantly more likely to have attended formal training in health management, they rated themselves significantly lower than private sector managers, and generally as being only "reasonably competent but not good" in all of the competencies. It is therefore reasonable to infer that formal programs currently in existence are either inappropriate or do not fully meet the needs of hospital managers in the public sector. This may also partly explain why they were significantly more likely to report that they were unprepared for their current responsibilities and were more likely to want to seek further training in management. This supports the finding of Pillay [15], that formal management development programs did not significantly enhance competency levels of health managers. Instead they appeared to derive greater benefit from more informal approaches which include mentoring, coaching and in-house approaches.

It is important to note that ranking of these competencies by managers was purely subjective and based on a self-assessment, which was not externally validated. It may have been influenced by the respondents lack of knowledge with the topic and therefore a lack of confidence in being able to rate the items, or it may have been based on a self-evident knowledge gap. The competencies listed may also not have fully reflected the scope of hospital management. However, despite these limitations, the study has important theoretical and practical relevance for the improvement of health management capacity in South Africa.

## Conclusion

In conclusion, the findings confirm our supposition that there is a lack of management capacity within the public sector in South Africa and that there is a significant gap between private and public sectors. It provides the evidence that there is a great need for the further development of managers, especially those in the public sector. The onus is therefore on administrators and those responsible for management education and training to identify managers in need of development and to make available training that is contextually relevant in terms of program design and delivery.
